# Mapping LUCAS topsoil chemical properties at European scale using Gaussian process regression

**DOI:** 10.1016/j.geoderma.2019.113912

**Published:** 2019-12-01

**Authors:** Cristiano Ballabio, Emanuele Lugato, Oihane Fernández-Ugalde, Alberto Orgiazzi, Arwyn Jones, Pasquale Borrelli, Luca Montanarella, Panos Panagos

**Affiliations:** a European Commission, Joint Research Centre (JRC), Ispra, Italy; b Environmental Geosciences, University of Basel, Switzerland

**Keywords:** pH, Cation Exchange Capacity, Nitrogen, Phosphorus, Potassium, C:N ratio, Mapping, Gaussian process regression

## Abstract

This paper presents the second part of the mapping of topsoil properties based on the Land Use and Cover Area frame Survey (LUCAS). The first part described the physical properties (Ballabio et al., 2016) while this second part includes the following chemical properties: pH, Cation Exchange Capacity (CEC), calcium carbonates (CaCO_3_), C:N ratio, nitrogen (N), phosphorus (P) and potassium (K). The LUCAS survey collected harmonised data on changes in land cover and the state of land use for the European Union (EU). Among the 270,000 land use and cover observations selected for field visit, approximately 20,000 soil samples were collected in 24 EU Member States in 2009 together with more than 2000 samples from Bulgaria and Romania in 2012. The chemical properties maps for the European Union were produced using Gaussian process regression (GPR) models. GPR was selected for its capacity to assess model uncertainty and the possibility of adding prior knowledge in the form of covariance functions to the model.

The derived maps will establish baselines that will help monitor soil quality and provide guidance to agro-environmental research and policy developments in the European Union.

## Highlights

• The LUCAS harmonised soil survey comprising 20,000 observations was used in this study.
• Soil chemical properties were mapped over the extent of Europe using Gaussian process regression (GPR).
• GPR with Matérn kernel modelled soil properties with high accuracy.
• GPR allows the production of uncertainty maps as it allows the estimation of prediction variance.
• These maps complete the series of topsoil properties for Europe based on LUCAS.

## 1 Introduction

Globally, soil and environmental challenges (climate change, pollution, water scarcity, biodiversity decline) are increasing dramatically (IPBES, 2019). Organizations such as the United Nations Convention to Combat Desertification (UNCCD), the Food Agriculture Organization (FAO) and the Intergovernmental Science-Policy Platform on Biodiversity and Ecosystem Services (IPBES) have recognized that soil is under threat globally (Montanarella, 2015). The development of solutions to combat land degradation requires data collection, expert knowledge, and scenario analysis through the modelling of soil properties and functions (Hartemink, 2015). During the past two decades, the soil science community has developed regional, continental and worldwide soil maps and databases, which have been used for soil resource assessment and risk evaluation (Arrouays et al., 2017).

During the past decade, the increased use of digital soil mapping (McBratney et al., 2003) approaches became a solution to increased requests for spatial soil data coming from research organizations, policy makers and the private sector. The extensive development of digital soil mapping models has been facilitated by the exponential increase in the availability of remote sensing data, computing power, and the development of Geographic Information Systems (Minasny and McBratney, 2016). Digital soil mapping has important advantages in the prediction of soil properties (e.g. evidence-based, open access data and software, transparent and repeatable methodology, etc.) compared to conventional soil mapping approaches (Minasny et al., 2018). Among others, Grundy et al. (2015) mapped 11 soil properties in Australia using a geostatistical approach, Padarian et al. (2017) contributed to the development of a Global Soil Map by modelling eight properties for Chile, Mansuy et al. (2014) generated national maps of soil properties for managed forests in Canada, Adhikari et al. (2014) developed soil organic carbon content maps for Denmark while Poggio and Gimona (2014) modelled soil organic carbon stocks in Scotland.

In the European Union (EU), Ballabio et al. (2016) have developed physical properties datasets (silt, clay, sand and coarse fragments) for the EU, together with maps of derived products (bulk density, available water capacity) using the Land Use and Cover Area frame Survey (LUCAS) topsoil database. In addition, de Brogniez et al., 2015 and Yigini and Panagos, 2016 have mapped the soil organic carbon content (expressed as percent and carbon stocks) using LUCAS topsoil database and geostatistical models.

The objective of this paper is to produce chemical soil properties datasets using LUCAS topsoil database and advanced digital soil mapping methodologies. In detail, this paper proposes a soil mapping model for interpolating the 22,000 surveyed points in LUCAS for chemical soil properties at continental scale. Finally, we developed datasets for soil pH, Cation Exchange Capacity (CEC), calcium carbonates (CaCO_3_), and total phosphorus, potassium and nitrogen, plus derived products based on soil organic carbon and nitrogen (e.g. C:N ratio) covering the 26 EU Member States (excluding Croatia and Cyprus).

It is not the objective of the paper to challenge any local or regional map of chemical properties implemented with higher density of surveyed points. Moreover, this paper will provide only a cursory explanation for the reasons for different spatial patterns on chemical properties as our focus is the digital soil mapping model development.

## 2 Mapping chemical properties at European scale

This section gives an overview of existing activities relevant to chemical properties using digital soil mapping. Soil chemical properties have a spatial dependence; their spatial patterns depend on soil forming processes (including climate, parent material and weathering), topography, climate, vegetation, time and anthropogenic influences (Yost et al., 1982).

**Soil pH** is dependent on the parent material, climate and soil organic carbon. Where precipitation levels are high, soil pH decreases over time through acidification due to leaching of base cations and corresponding build-up of hydrogen ions. In dry environments, where chemical weathering and leaching are less intense, soil pH may be neutral or alkaline, as a result of evaporation of alkaline groundwater. In general higher rainfall rates result in acid soils and the water balance seems to act globally as a main driving factor for soil pH (Slessarev et al., 2016), while topography and mineralogy may act as secondary drivers. At European scale, soil pH datasets have been produced by the Forum of European Geological Surveys (FOREGS) (Salminen et al., 2005) using 1588 soil samples across 28 countries. The project Geochemical Mapping of Agricultural and Grazing Land Soil in Europe (GEMAS) sampled 2200 points in agricultural land and 2118 points in permanent grasslands in 2008–2009. In 2012, a harmonised pH dataset was compiled by the JRC and disseminated through the European Soil Data Centre (ESDAC) (Panagos et al., 2012). The ESDAC pH dataset is based on 12,333 measurements from 11 different data sources which is a significant limitation, due to heterogeneity in the measurements, compared to the LUCAS soil sampling scheme. The recent publication of SoilGrids (Hengl et al., 2014) was an important advancement for soil pH data availability at global scale.

**Calcium carbonates** are derived from the weathering of lime-rich parent material (Lal, 2007). Soil carbonates, most commonly represented by calcium carbonate, have multiple functions in soils. Firstly, they help slowing soil acidification by acting as a buffer to increased levels of aluminium and hydrogen ions, thus also preventing the uptake of heavy metals by plants. Another important function is the stabilization and the improvement of both soil organic carbon content and soil structure. Sarmadian et al. (2010) used geostatistical methods to produce a topsoil calcium carbonates map. At continental scale, Wilford et al. (2015) predicted soil calcium carbonate concentrations in Australia using data from 1311 sites. According to Wilford et al. (2015), the key predictors of CaCO_3_ in Australia include mean annual precipitation, mean annual radiation, soil types, mean annual temperature, and the MODIS vegetation coefficient of flatness.

**Cation Exchange Capacity** (CEC) is often considered as an indicator of soil quality and measures the ability of soil to hold and exchange cations (Saidi, 2012). CEC is related to stable aggregates and texture properties (Bronick and Lal, 2005). CEC is often estimated using pedotransfer rules or other algorithms such as artificial neural networks (Amini et al., 2005). Few studies have estimated CEC at country scale and, among them, Khaledian et al. (2017) derived correlations between CEC and physical attributes (clay, silt, sand) and chemical attributes (pH). Bishop and McBratney (2001) applied different geostatistical models to estimate the CEC in northern New South Wales, Australia.

**Nitrogen** (N) spatial distribution is not only affected by natural ecological processes, but also impacted by intensive human activities (K. Wang et al., 2013). This is an important challenge for accurate predictive mapping at regional scales. The C:N (organic carbon to nitrogen) ratio is an index of the organic matter turnover and nitrogen availability due to mineralization or immobilization of soil nitrogen. Using more than 4000 soil profiles around the world from the World Inventory of Soil Emission Potentials (WISE) database, Batjes (1996) estimated a wide range of mean C:N ratios for the 0–30 cm topsoil starting from 9.9 for arid Yennosols to 25.8 for Histosols.

**Phosphorus** (P) concentrations are also influenced by human activity. Fertilization can result in higher levels of P, especially in higher yields crops where high input of P fertilizers are reported (Tóth et al., 2014). Modern agriculture is much dependent on phosphorus fertilizers, and P supply is strategically critical at global level. Using a geostatistical model, Rossel and Bui (2016) mapped phosphorus stocks in Australia at approximately 90 m resolution.

**Potassium** (K) has different functions for plant life; it is a constituent of enzymes and acts as a regulator of drought tolerance and water use (M. Wang et al., 2013). In the soil, the principal sources of potassium are feldspars and micas, which release K during weathering (Hillel, 2008). Potassium depletion from soil is quite uncommon as cation exchange prevents leaching. Few studies have mapped potassium at continental scale; among them, Prado et al. (2008) mapped the potassium distribution in Brazil using a limited amount of data and extrapolated it to the whole country.

## 3 Material and methods

This section describes the main data input which is the LUCAS topsoil database, how it has been compiled based on the survey and the laboratory analysis.

### 3.1 Land Use/Land Cover Area Frame Survey (LUCAS) topsoil database

The Land Use/Land Cover Area Frame Survey (LUCAS) is a project to monitor land use and land cover changes across the EU. The LUCAS survey is performed every three years, with the latest published LUCAS dataset dating back to 2015. It now covers all the 28 EU countries and includes field observations at more than 273,000 points. Soil samples are taken in about 10% of the surveyed locations every 6 years. The first LUCAS soil survey was done in 2009 collecting 19,969 topsoil samples (0–20 cm) from 25 out of 28 EU countries, excluding Romania, Bulgaria and Croatia (Orgiazzi et al., 2018). In the 2012 LUCAS survey, 2034 topsoil samples were collected from Bulgaria and Romania following the standard procedure of 2009. The overall sampling density of this pan-European soil survey is nearly one soil sample every 196 Km^2^ (Panagos et al., 2013), which means one sample about every 14 km × 14 km. In this paper, we used the chemical properties based on 2009–2012 LUCAS topsoil as the analysis of the 2015 soil samples is still ongoing.

The LUCAS topsoil dataset is the most comprehensive and harmonised soil dataset at European scale, which allows pan-EU studies on the distribution of physical properties (clay, silt and sand) (Ballabio et al., 2016), soil erodibility (Panagos et al., 2014), soil organic carbon (de Brogniez et al., 2015) and the modelling of heavy metals diffuse pollution such as copper (Ballabio et al., 2018). The number of points selected is based on a stratification in order to cover all possible land uses (based on CORINE land cover classes) and country surface (Carre et al., 2013). Orgiazzi et al. (2018) described in detail the soil sampling procedure and the standards that the surveyors should follow. The soil samples were analysed for the percentage of coarse fragments, particle-size distribution (silt, clay, sand), pH, organic carbon, calcium carbonate, soluble phosphorous, total nitrogen, extractable potassium, Cation Exchange Capacity (CEC) and multispectral properties (Tóth et al., 2013a, Tóth et al., 2013b). Due to problems in labelling, tagging, geo-referencing and mismanagement, 321 soil samples were excluded from LUCAS topsoil database, resulting in 21,682 total records.

### 3.2 Laboratory analysis of soil samples for chemical properties

The sample analysis was performed by a single laboratory, contributing to data comparability avoiding uncertainties due to analysis based on different methods or different calibrations in case of multiple laboratories. In a first phase, LUCAS topsoil samples were analysed for their physical and chemical properties following ISO standard procedures. In a later stage, an additional analysis for heavy metals was performed.

### 3.3 Auxiliary variables

To support the spatial predictions of soil properties, a series of datasets or covariates were selected according to their possible influence on soil chemical properties. The spatial resolution of the covariates was set to 250 m, as a compromise between the resolution of the Moderate-resolution Imaging Spectroradiometer (MODIS) data (500 m), the finer resolution of the DEM (25 m) and the coarser WorldClim climatic (1 km) datasets (Fick and Hijmans, 2017). Overall 100 numeric and 99 dummy covariates were considered in the first steps of the analysis. The dummy covariates were obtained from the coding of the categorical variables classes (CORINE and parent material type) into dichotomous variables.

After feature selection, the name and a description of the covariates retained in the final model is given in Table 1.

**Table 1:** Naming and description of the covariates used in the models (the covariate code corresponds to the one used in the variable relevance plots of Fig. 2**).**

Covariate code	Description
mir_PCAb*i*	Component *i* of PCA transformed of MODIS multitemporal Mean Infrared band for year 2009
nir_PCAb*i*	Component *i* of PCA transformed of MODIS multitemporal Near Infrared band for year 2009
red_PCAb*i*	Component *i* of PCA transformed of MODIS multitemporal Red band for year 2009
blue_PCAb*i*	Component *i* of PCA transformed of MODIS multitemporal blue band for year 2009
pheno_MODIS_LAEA.1	Periodic component *i* of MODIS NDVI time series Fourier harmonic analysis
trend_MODIS_LAEA.1	Trend component *i* of MODIS NDVI time series Fourier harmonic analysis
tmax*i*_500	Average max temperature of month *i* from WorldClim
tmin*i*_500	Average min temperature of month *i* from WorldClim
prec*i*_500	Average precipitation of month *i* from WorldClim
bio*i*_500	Bioclimatic index *i* from WorldClim
y	Latitude
x	Longitude
elevation	Elevation
valley height	Valley height index
gen_surface	Smoothed Elevation
ls	RUSLE topographic factor (Slope Length and Steepness factor)
aacn	Altitude above channel network
airflow_height	Effective Air Flow Heights (Böhner and Antonić, 2009)
downsl_dist_grad	Downslope Distance Gradient (Hjerdt et al., 2004)
corine.*i*	Class *i* of CORINE land cover
geo.*i*	Class *i* of ESDB parent material

Cyprus was excluded from the analysis due to missing covariates.

#### 3.3.1 MODIS and derived data

A series of MODIS image products for 2009 was collected; in particular, the MODIS Global vegetation indices (Didan, 2005). These products are characterised by a spatial resolution between 250 and 500 m and a temporal resolution of 16 days. The products include blue, red and near- and mid-infrared reflectance, centered at 469 nm, 645 nm, and 858 nm respectively. The reflectance is used to determine the MODIS daily vegetation indices, such as the Normalized Difference Vegetation Index (NDVI) and the Enhanced Vegetation Index (EVI). NDVI is defined as *NDVI* = (*NIR* − *RED*)/(*NIR* + *RED*), where NIR and RED stand for the spectral reflectance measurements acquired in the near-infrared and visible (red) regions, respectively. NDVI has been used to estimate a large number of vegetation properties from its value, such as biomass, chlorophyll concentration in leaves, plant productivity, fractional vegetation cover and accumulated rainfall.

The EVI index is defined as:

(1) $$\mathrm{EVI}=g∙\frac{\mathrm{NIR}-\mathrm{RED}}{\mathrm{NIR}+c1∙\mathrm{RED}-c2∙B\mathrm{LUE}+L}$$

where NIR, RED, and BLUE are the respective surface reflectance in the corresponding spectral bands, *L* is the canopy background adjustment, and *c*1 and *c*2 are coefficients for the aerosol resistance term, which uses the blue band to correct for aerosol influences on the red band. The coefficients adopted by the MODIS-EVI algorithm are: *L* = 1, *c*1 = 6, *c*2 = 7.5, and *g* (gain factor) = 2.5.

Phenological indices were derived from MODIS data using a first order harmonic model on the EVI and NDVI multi-temporal data. The harmonic uses a discrete Fourier processing that decomposes temporal curves in a linear trend plus amplitude, variance and phase metric terms. The harmonic model can be defined as

(2) $$\hat{Y_{t}}=\alpha_{0}+\sum_{j=1}^{m}\alpha_{j}\;\cos\left(\frac{j2\mathrm{\pi t}}{l}\right)+\beta_{j}\;\sin\left(\frac{j2\mathrm{\pi t}}{l}\right)$$

where ${\hat{Y}}_{t}$ is the vegetation index value, *t* is the time value for a given pixel, *l* is the cycle length (yearly) and *m* is the order of the trigonometric polynomial and coincides with the number of harmonics of the expansion (set as one in this study), *α*_*j*_ and *β*_*j*_ are the Fourier coefficients.

Harmonic analysis using Fourier series, has been used to model the temporal changes in the vegetation cover using satellite data for several decades (Menenti et al., 1993; Moody and Johnson, 2001; Olsson and Eklundh, 1994) and provides better spatial information on the different types of vegetation cover than using composite images alone.

Additionally, a Principal Component Analysis (PCA) transformation of the full MODIS 16 day images time series was performed for each band in order to extract relevant features. The PCA projects the time correlated input images into uncorrelated PCA components ordered according to their variance. Thus, the first few components account for most of the time related variation in each MODIS band.

#### 3.3.2 Terrain parameters

The EU-DEM digital elevation model (Bashfield and Keim, 2011) was used to derive land features at a resolution of 25 m for all Europe.

Both the DEM and the derived surface parameters were then rescaled to 250 m. The derivation of land surface parameters was made using the SAGA GIS software. Among the various parameters derived and tested, the most relevant were the Multi-resolution Valley Bottom Flatness (MRVBF) and the Multi-resolution Ridge Top Flatness (MRRTF) (Gallant and Dowling, 2003), slope, slope height and vertical distance to channel network (CNBL).

#### 3.3.3 Land cover

The CORINE (CORdinate INformation on the Environment) is a raster format land cover database comprising 44 classes. CORINE is derived from Earth observation satellites using computer-aided photointerpretation. The nominal scale of CORINE is 1:100,000 with a minimum mapping unit (MMU) of 25 ha and a change detection threshold of 5 ha. The CORINE dataset was used to represent the spatial distribution of land use and land cover. The reliability of CORINE 2000 version at 95% confidence level is 87.0 ± 0.7%, according to the independent interpretation performed on the LUCAS (Land Use/Cover Area Frame Survey) data (Büttner, 2014).

#### 3.3.4 Climate data

Monthly temperature averages and extremes, and monthly average precipitation values were obtained from the WorldClim (http://www.worldclim.org/) dataset at a spatial resolution of 1 km^2^. These data layers are the interpolated values of average monthly climate data collected from numerous weather stations. The approach uses a thin plate smoothing spline with latitude, longitude and elevation as independent variables to locally interpolate data (Hijmans et al., 2005). Climatic data was included explicitly in the model in the form of monthly values of minimum and maximum temperature and monthly rainfall rates. Also the bioclimatic variables (Temperature and precipitation indexes) of WorldClim were included in the analysis. Given the high collinearity of climate data, a careful feature selection procedure was applied in the model training stage.

#### 3.3.5 Legacy soil data and parent material geochemistry

In the first stage of this study, the European Soil Database (ESDB) (Panagos et al., 2012) was considered as a possible covariate to characterise soil properties. In this context, the ESDB was utilised as a multinomial variable by identifying and labelling soil types. However, the use of the ESDB soil data was found to provide little improvement to the model outcome and was then removed from the analysis. Nonetheless, the data within the ESDB was used to create a map of the parent material geochemistry that was included in the model.

### 3.4 Gaussian process regression models for chemical properties

In order to assess the relation between environmental features and soil chemical properties distribution, Gaussian Process Regression (GPR) (Rasmussen and Williams, 2006) was utilised for inference and mapping.

GPR assumes that the output *y* of a function *f* with input x can be expressed as

(3) $$y=f\left(x\right)+\epsilon$$

where $\epsilon \sim N\left(0,\sigma_{\epsilon }^{2}\right)$. This is analogous to linear regression. However in GPR, not only the error term *ϵ*, but also *f* is treated as a random variable. In GPR, *f*(x) is distributed as a Gaussian process

(4) $$f\left(x\right)\sim \mathrm{GP}\left(\mu \left(x\right),k\left(x,x^{*}\right)\right)$$

where *f*(x) is defined by its mean *μ*(x) and covariance *k*(x, x^∗^).

The covariance function *k* is also known as the kernel of the GPR and models the dependence of the function values between different values of x. In this respect, GPR is equivalent to kriging (Stein, 2012); however, while kriging is usually performed in geographical space, GPR is applied on an arbitrary number of covariates. The choice of the appropriate kernel is based on the structure, in terms of smoothness and peculiar patterns, of the data itself.

In this study, the Matérn kernel function (Stein, 2012) was used. The Matérn function is quite flexible as it can model data with different smoothness; the function can be written as

(5) $$k\left(x,x^{*}\right)=\frac{2^{1-\nu }}{\Gamma \left(\nu \right)}{\left(\frac{\sqrt{2\nu }}{\ell }\left|x-x^{*}\right|\right)}^{\nu }B_{\nu }\left(\frac{\sqrt{2\nu }}{\ell }\left|x-x^{*}\right|\right)$$

where *ν* and *ℓ* are positive adjustable parameters, *B*_*ν*_ is a modified Bessel function of the second kind of order *ν* and Γ is the Gamma function. The *ℓ* acts as a scale parameter, while *ν* controls the process smoothness. In general, values of *ν* are kept within the range between 1/2, where the process becomes rough, and 7/2, where it becomes difficult to distinguish between finite values of *ν* and *ν* → ∞.

The kernel function is equivalent to a covariance function in kriging and its value can be considered as a measure of similarity between the two feature vectors.

GPR can be seen as a Bayesian Nonparametric approach to regression, where the function from the Gaussian processes takes values in a (possibly infinite) function space. Defining y as the vector of observed values of the dependent variable and X as the matrix of the corresponding covariates and defining y^∗^ as a set of points to be predicted with the corresponding matrix of covariates X^∗^, a random vector can be drawn from the join prior distribution of functions as

(6) $$y^{*}\sim N\left(0,K\left(X^{*},X^{*}\right)\right)$$

where *K*(X^∗^, X^∗^) is the covariance matrix between inputs at points to be predicted. However, any vector drawn from the prior will provide no knowledge about the observed data. In order to get the posterior distribution over functions, the joint prior distribution must be restricted to contain only those functions which agree with the observed data. So given that the joint distribution of y and y^∗^ is

(7) $$\left[\begin{array}{c} y\\ y^{*} \end{array}\right]\sim N\left(0,\left[K\left(X,X\right)+\sigma_{n}^{2}IK\left(X,X^{*}\right),K\left(X,X^{*}\right)K\left(X^{\prime },X^{*}\right)\right]\right)$$

(where *K*(X, X) is the covariance matrix between all observed points inputs (covariates), *K*(X, X^∗^) is the covariance between observed points and points to be predicted and *σ*_*n*_^2^I is the identity matrix multiplied by the estimated (or presumed) variance of the observations) predictions for the new points y^∗^ corresponding to the covariates matrix X^∗^ can be derived as

(8) $$y^{*}=K\left(X^{*},X\right){\left[K\left(X,X\right)+\sigma_{n}^{2}I\right]}^{-1}y$$

and the variances for the elements of y^∗^ can be obtained from the diagonal of the covariance matrix COV(y^∗^)

(9) $$\mathrm{COV}\left(y^{*}\right)=K\left(X^{*},X^{*}\right)-K\left(X^{*},X\right){\left[K\left(X,X\right)+\sigma_{n}^{2}I\right]}^{-1}K\left(X,X^{*}\right)$$

Moreover, as the posterior distribution of Eq. (7) can be rewritten as

(10) $$\mu \left(x\right)=\sum_{i=1}^{t}w_{i}k\left(x,x_{i}\right)$$

(where x_*i*_ is an observed value in X**,** and the weights come from the vector w = [*K*(X, X) + *σ*_*n*_^2^I]^−1^y), GPR is effectively equivalent to a linear regression where inputs are projected into an higher dimensional space using basis functions (the kernel), predictions are then obtained by weighting the input values to the input values of the point to be predicted. In this manner GPR retains the conceptual simplicity of linear regression while having the capability of fitting arbitrarily complex relations of machine learning approaches.

An advantage of GPR over other machine learning approaches is that the process models both the expectation and the variance of the random variable, thus allowing mapping the prediction uncertainty. Moreover, the GPR allows the specification of the input data noise, so if prior knowledge about it is known, it can be used to avoid overfitting the data.

Another advantage of using GPR is that any linear combination of kernels is itself a valid kernel. This property can be used to model data that has different scale dependent patterns (Ballabio and Comolli, 2010) or are a composition of periodic and trend components. In this study, a single and a composition of two Matérn kernels were tested. Since the composition requires the tuning of more parameters, *ν* was kept constant across kernels in the composition. While the composition performed generally better than the single kernel, the gain was not significant enough as to justify the extra tuning time required. Therefore, we applied the GPR using a single kernel.

While GPR is a powerful technique, its main drawback is its computational complexity. Given that the computational burden scales as$\;O\left(N^{3}\right)$ for model fitting, $O\left(N\right)$ for model prediction and $O\left(N^{2}\right)$ for variance prediction (where *N* is the number of observations) the number of covariates is usually kept as small as possible. The computational scaling is especially problematic in spatial mapping where the number of predictions is easily in the order of millions of raster cells. To ease this issue it is possible to use the Nyström kernel matrix approximation (Drineas and Mahoney, 2005) to compress matrices to a more manageable size. Moreover, it is advisable to use massive parallel processing in order to split the raster data into more manageable subsets as the prediction task is easy to parallelize.

The GPR models parameters were tuned using a repeated k-fold cross-validation, with k = 10, in order to avoid overfitting. Moreover, model performance was also evaluated using the same procedure. Models selection was performed by Simulated Annealing (SA) (Kirkpatrick et al., 1983) in order to select the best set of covariates and thus reducing the chance of collinearity. SA was also utilised to estimate the relative information value of each covariate. As the selection is based on the reduction in the k-fold cross-validation error estimates, changes in the error due to a given predictor being added or removed from the model are tracked during the SA. This track of error changes is then aggregated as the variable information value.

## 4 Results

### 4.1 Model performance

In this study, GPR is quite efficient in predicting soil chemical properties with values of R^2^ ranging from 0.91 to 0.35. In general, the properties more related with the vegetation cover (nitrogen content, pH) are the most successfully modelled, while properties such as CEC result in higher errors as measured by Root Mean Squared Error (RMSE) and Mean Absolute Error (MAE). This is likely due to the influence of other factors (e.g. soil clays mineralogy, soil age, etc.), that have little or no direct connection with the covariates included in this study. In particular, for soil age and mineral weathering, there are no available covariates that could be used at this scale. Moreover, properties such as CEC are inherently difficult to measure precisely, making their modelling subject to a higher noise to signal ratio.

**Fig. 1:**
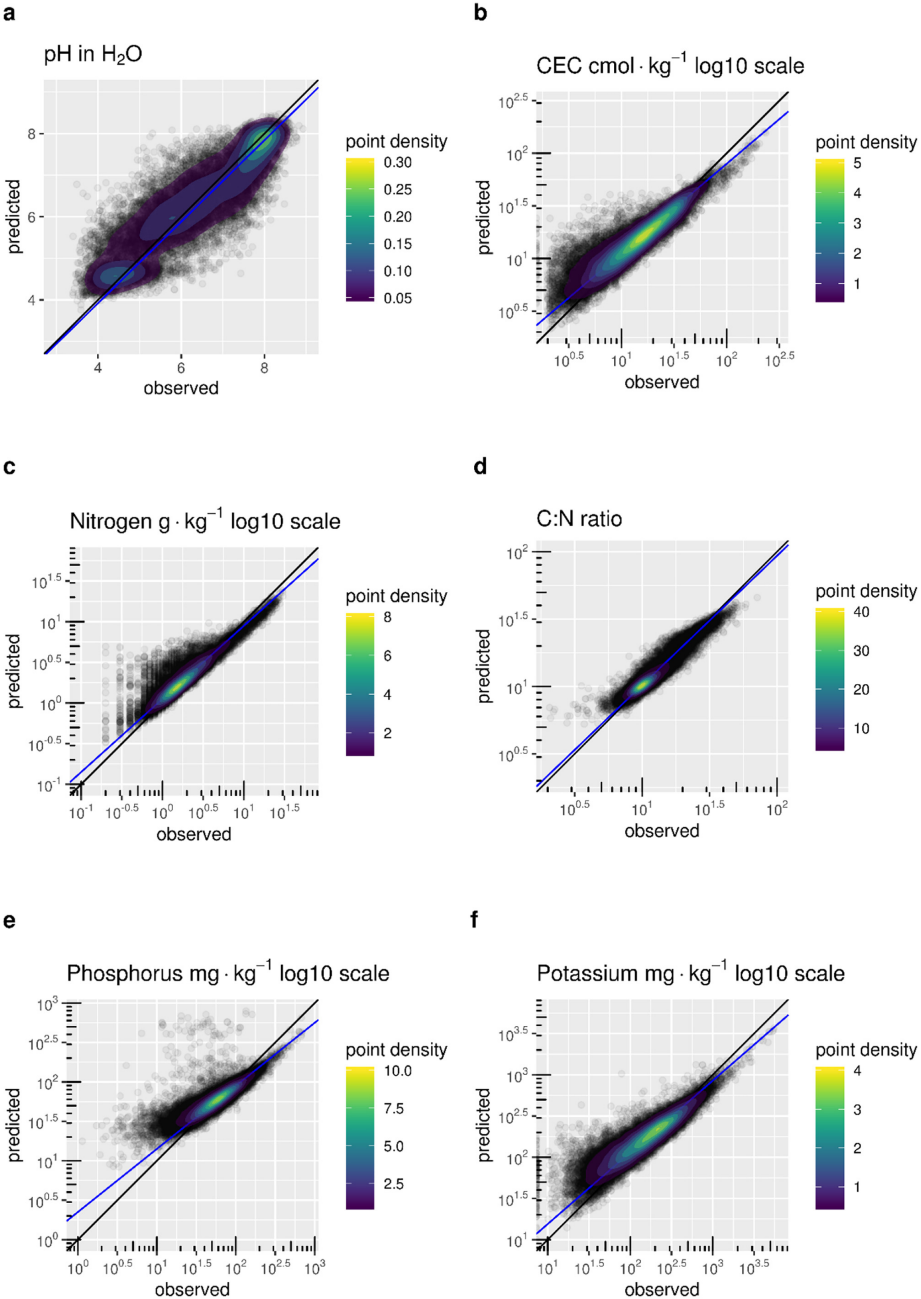
Predicted vs observed values for the topsoil properties discussed in Section 4.2. The blue line represent a linear fit for predicted-vs-observed data. The black line is the diagonal and the contours represent point densities. (For interpretation of the references to color in this figure legend, the reader is referred to the web version of this article.)

**Fig. 2:**
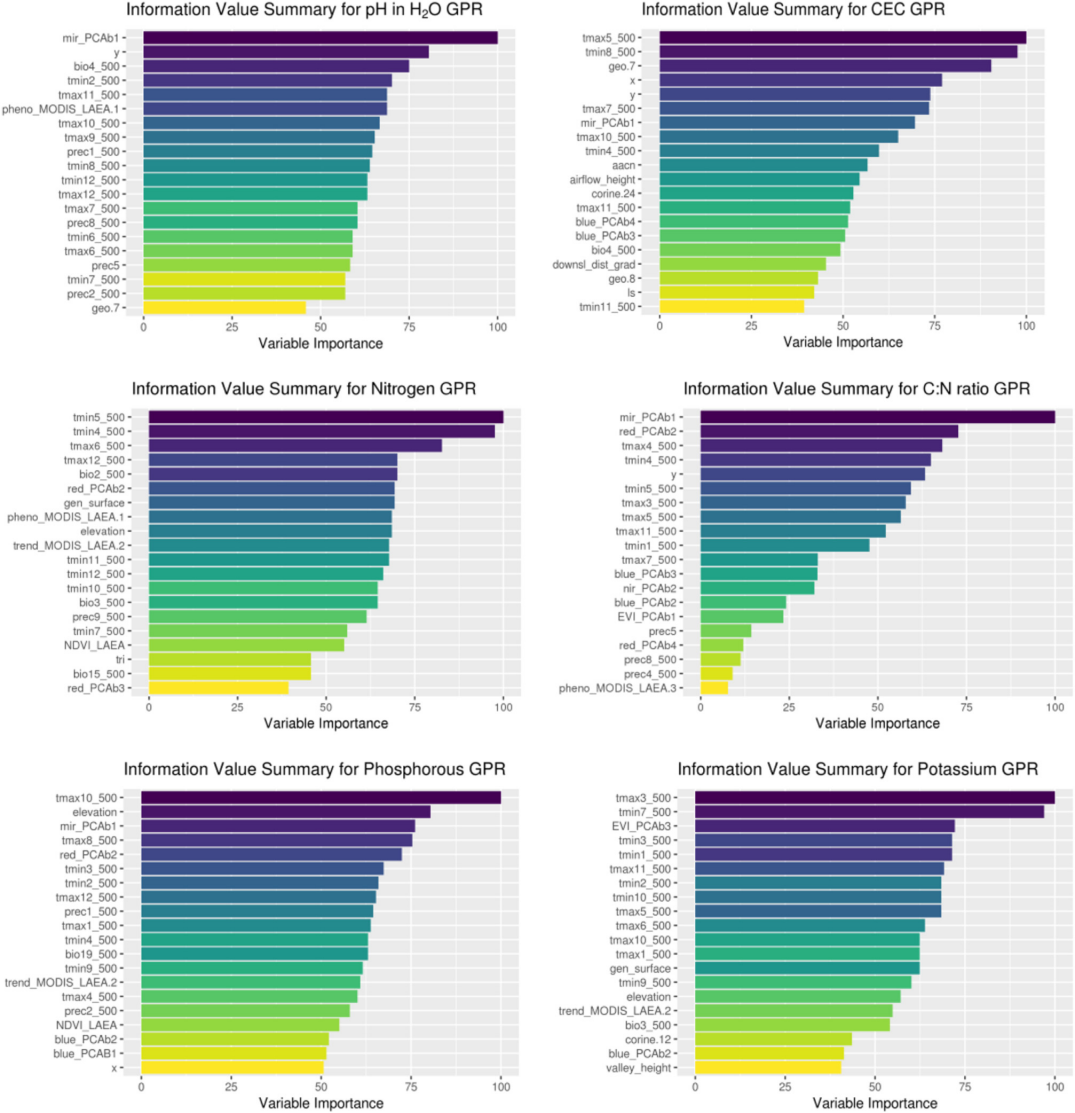
Variable importance metrics for the topsoil properties discussed in Section 4.2.

GPR regression performance metrics for chemical properties (Table 2) shows the performances of the models in terms of RMSE, MAE, Relative Squared Error (RSE) and R^2^; RMSE and MAE give error values in the measurement unit of the original variable, so their values can be compared with the mean and median values. RSE and R^2^ are metrics that can be used to compare the performance of the different models. The values were computed from repeated k-fold cross-validation. For maps discussed in Section 4.2 the plots of predicted vs observed values are shown in Fig. 1, while variable information values are shown in Fig. 2. The plots of Fig. 1 seem to suggest the presence of some prediction bias for several of the properties. However, the prediction bias is present only for extreme values. In general, values within the 95th percentile are quite well predicted with residuals normally distributed and showing absence of bias.

**Table 2:** GPR regression performance metrics for chemical properties. The mean and median values in LUCAS data are given as a reference.

	Mean	Median	RMSE	MAE	RSE	R^2^
CaCO_3_ g·kg^−1^	52.78	1.00	78.29	40.84	0.76	0.61
CEC cmol·kg^−1^	16.08	12.70	11.02	6.64	2.55	0.35
C:N ratio	13.20	10.89	1.97	1.26	0.12	0.91
Nitrogen g·kg^−1^	12.10	1.80	2.40	1.2	2.59	0.60
Phosphorous mg·kg^−1^	37.61	29.10	17.52	11.70	0.82	0.74
Ph in H_2_O	6.30	6.30	0.78	0.62	0.57	0.65
Ph in CaCl_2_	5.70	5.80	0.68	0.53	0.36	0.76
Potassium mg·kg^−1^	199.17	142.20	121.89	70.98	0.53	0.75

### 4.2 Topsoil chemical properties maps

This section will discuss some of the properties mapped, namely:

1. The topsoil pH in water and the difference between topsoil pH in water and in CaCl_2_ 0.01 M solution
2. The topsoil total nitrogen content and the topsoil C:N ratio
3. The topsoil total phosphorus content and the topsoil total potassium content

These properties were selected due to their distinctive spatial distribution and relevance in soil management.

#### 4.2.1 Topsoil pH

**Fig. 3:**
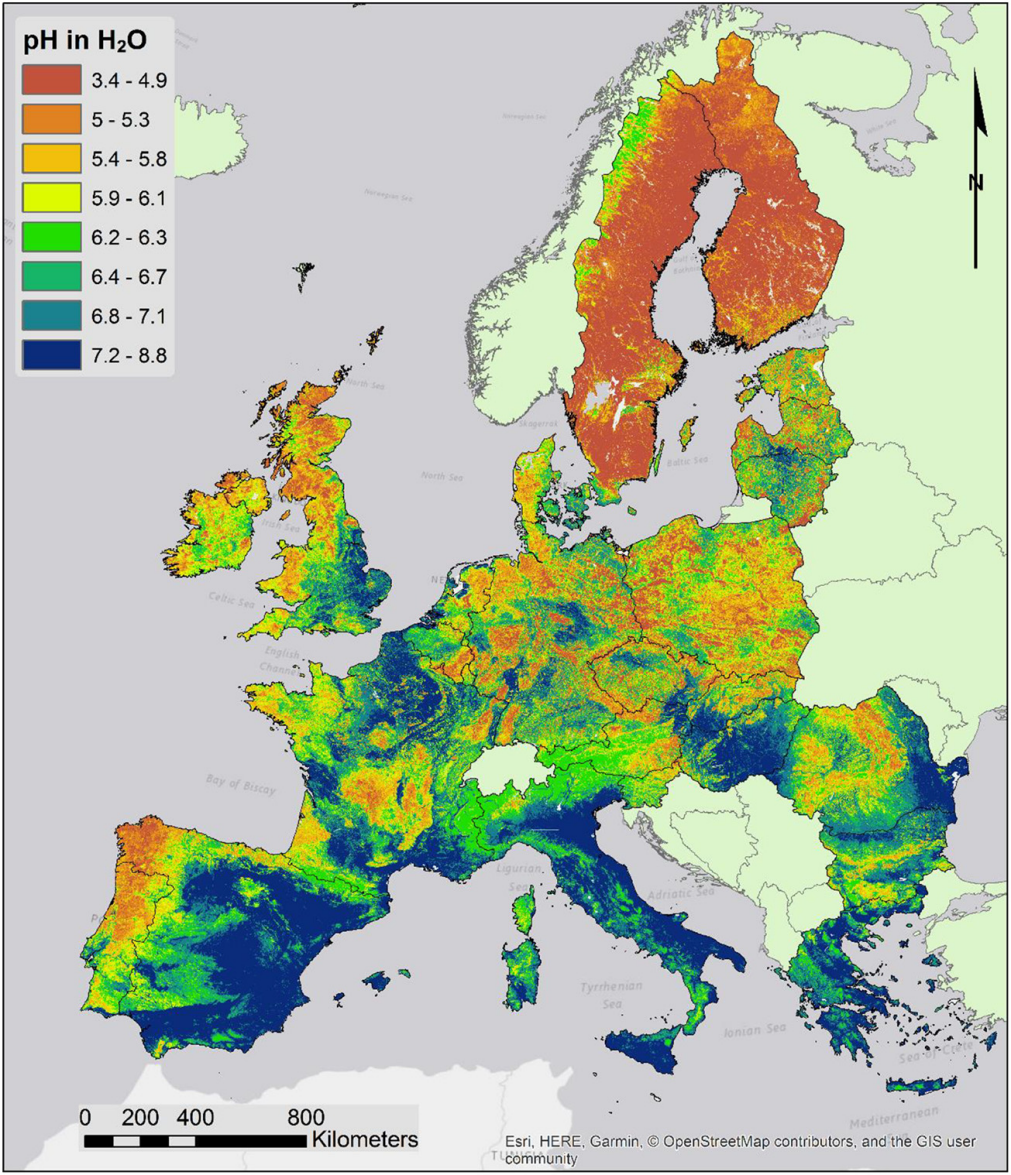
Map of topsoil pH in water.

The map of topsoil pH (Fig. 3) shows a clear influence of the geochemical makeup of soil parent material. Areas where carbonate rocks are present show higher pH levels; this is particularly obvious in areas where soil erosion can enhance the influence of the parent material such as in the area surrounding the Mediterranean Sea. In particular most of Spain, southern France, Italy and Greece have neutral to alkaline soils. The effect of geology is also quite evident in Northern France and most of United Kingdom. Nevertheless, climate can also influence pH and sometime overcomes the effect of the parent material. This is quite evident in Ireland and north-eastern Spain where rainfall is intense enough to leach the topsoil, resulting in acid soils. Vegetation cover is another factor influencing soil pH; in general forest cover, especially of coniferous species, tend to lower topsoil pH as they return fewer base cations to the soil with the litter. On the contrary, steppe grassland can result in increased topsoil pH as in the plains of Hungary where Chernozem soils typically have a topsoil enriched in calcium ions from the underlying loess deposits. The effect of vegetation is even more striking at shorter scales where the difference of pH between patches of forest and cultivated soils can be quite abrupt.

**Fig. 4:**
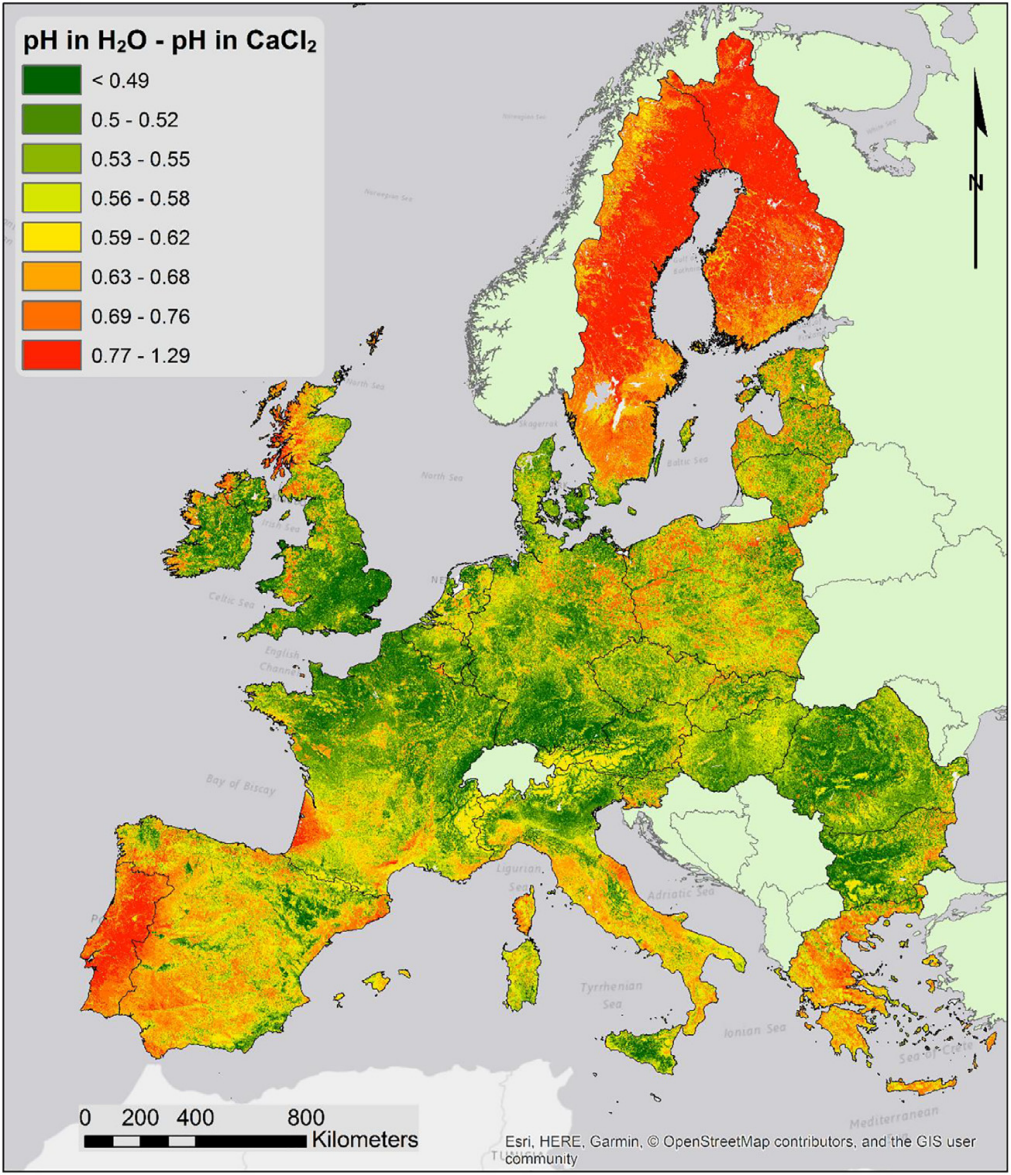
Map of the difference between pH in water and pH in CaCl_2_ 0.01 M solution.

The map of pH in CaCl_2_ solution (not shown) has a similar outline as the map of pH in water. Despite similarities the map of pH in CaCl_2_ is useful for determining the soil liming potential (Schofield and Taylor, 1955). Moreover, the difference between the two pH measures can give an idea of the exchangeable acidity of the topsoil (Fig. 4). Exchangeable acidity includes more or less ionized acid functions, weak organic acids and easily exchangeable cations. Commonly it is caused by clay hydrolysis, which results in some of Al^3+^ cations passing into exchangeable positions; this occurs naturally in some processes, like podsolization, but can be exacerbated by anthropic activities causing rain acidification or by acidifying fertilizers. As shown by Fig. 4, greater differences in pH values are found in areas where podsolization is the prevalent pedogenetic process, such as Scandinava and the Atlantic coast of Iberia and the west coast of France.

#### 4.2.2 Topsoil Cation Exchange Capacity

**Fig. 5:**
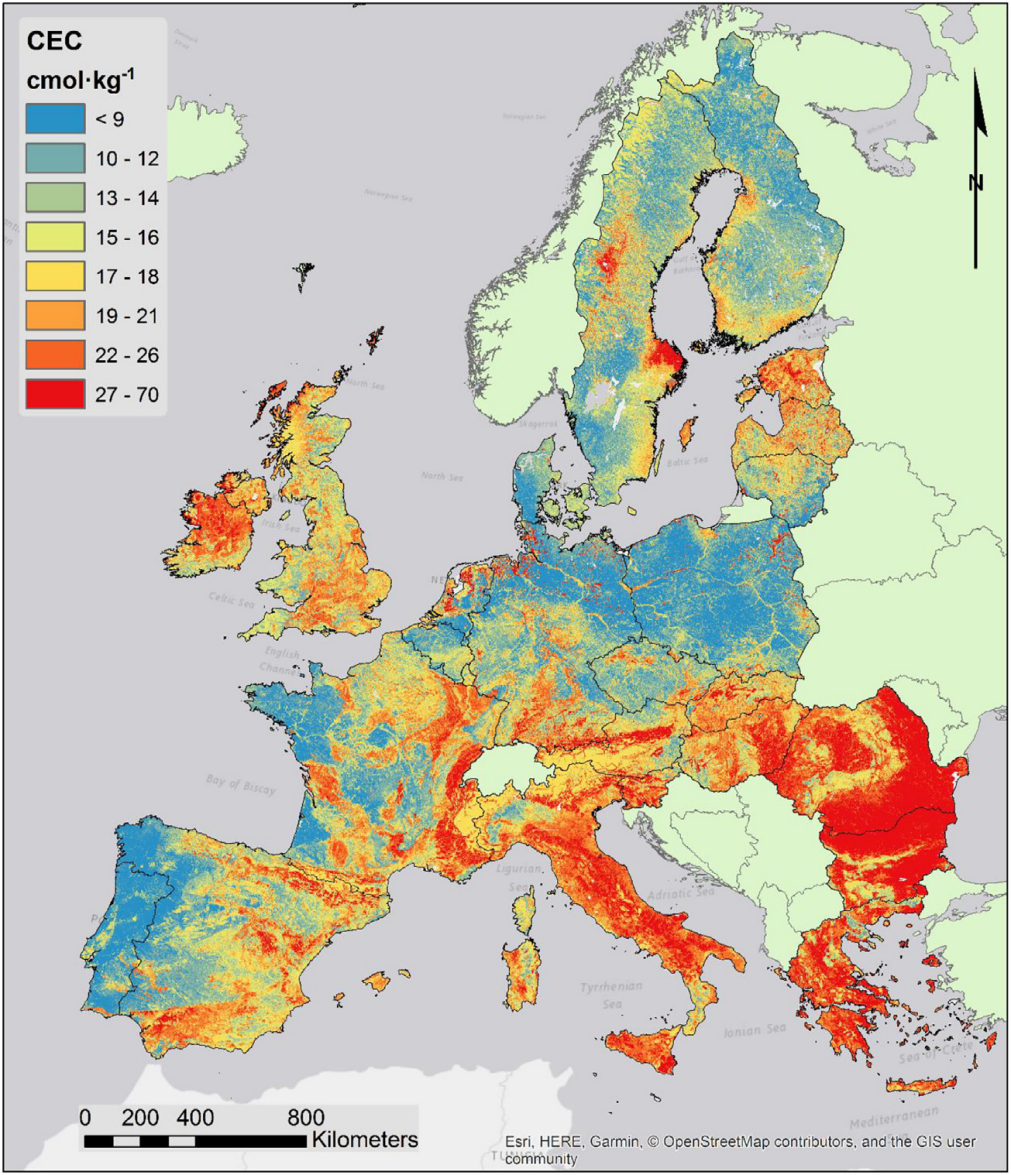
Map of topsoil CEC.

The map of CEC (Fig. 5) is mostly influenced by the distribution of clay in the topsoil. A comparison between clay distribution (Ballabio et al., 2016) and CEC shows many similarities. Remarkably, CEC is also quite influenced by topography, where areas of sediment accumulation have general higher values of CEC. This is quite visible in The Netherlands, Northern Germany and Poland, where the areas surrounding rivers have higher than average CEC. The geochemistry of the parent material also influences CEC. Besides limestone, where the relation with higher clay content is evident, soils developed on calcareous and marl rocks also tend to accumulate clay as a consequence of the leaching of the carbonates leaving the more stable clays behind.

#### 4.2.3 Topsoil nitrogen and C:N ratio

**Fig. 6:**
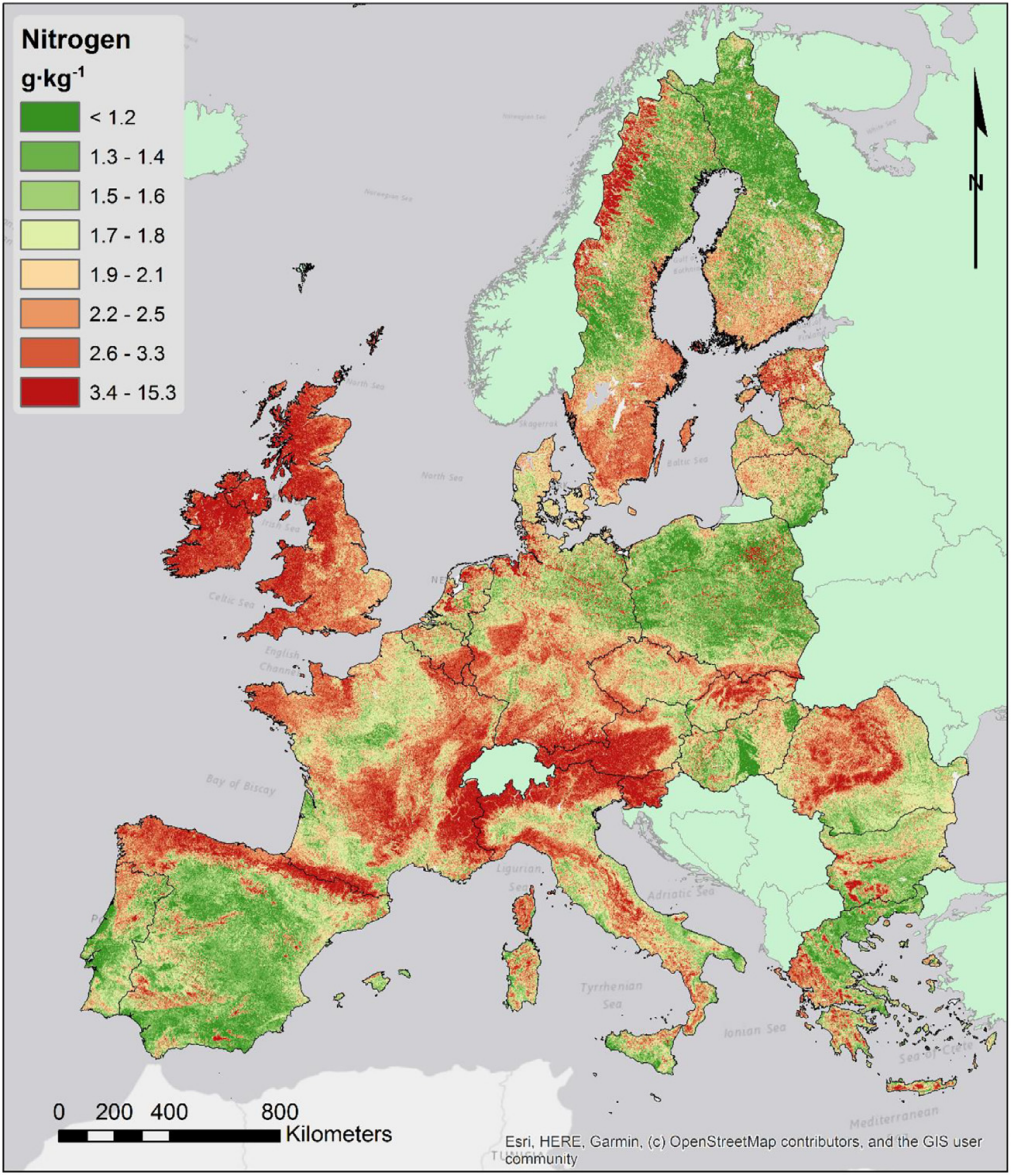
Map of topsoil nitrogen content.

The distribution of topsoil nitrogen (Fig. 6) is highly correlated with soil organic carbon, given that nitrogen is a main component of soil organic matter. While their ratio can vary, some carbon rich soils are also nitrogen rich, at least in terms of absolute quantities. Given this relation, it is quite clear that vegetation cover and climate are the main drivers in the distribution of nitrogen. As the map in Fig. 6 shows, forests and grasslands areas tend to have higher nitrogen content. Forests of Scandinavia, or those of the mountain areas are clearly outlined by the map. Climate also acts as a main driving force influencing nitrogen content along the Atlantic area; in particular, the United Kingdom and Ireland show higher N concentrations due to a fresh and humid climate which favours organic matter accumulation. Soil texture also plays a role in stabilizing organic matter and thus nitrogen. Areas with coarser soils, such as most of Poland, tend to have less nitrogen even if other conditions are favourable (e.g. vegetation, climate).

While the nitrogen concentration is relevant for assessing stocks and potential N_2_O emissions, the ratio between carbon and nitrogen can better represent the differences in the organic matter composition. Where higher rates correspond to more oligotrophic soils, typical of coniferous forests, or to peatland soils, lower rates are typical of more balanced nutrient-rich soils. Moreover, C:N ratio is a major determinant in the composition of the soil microbial community (Wan et al., 2015). However the C:N ratio is in turn influenced by the biota, with microbial dominated soils having lower C:N ratio than fungal dominated, in a typical feedback loop.

**Fig. 7:**
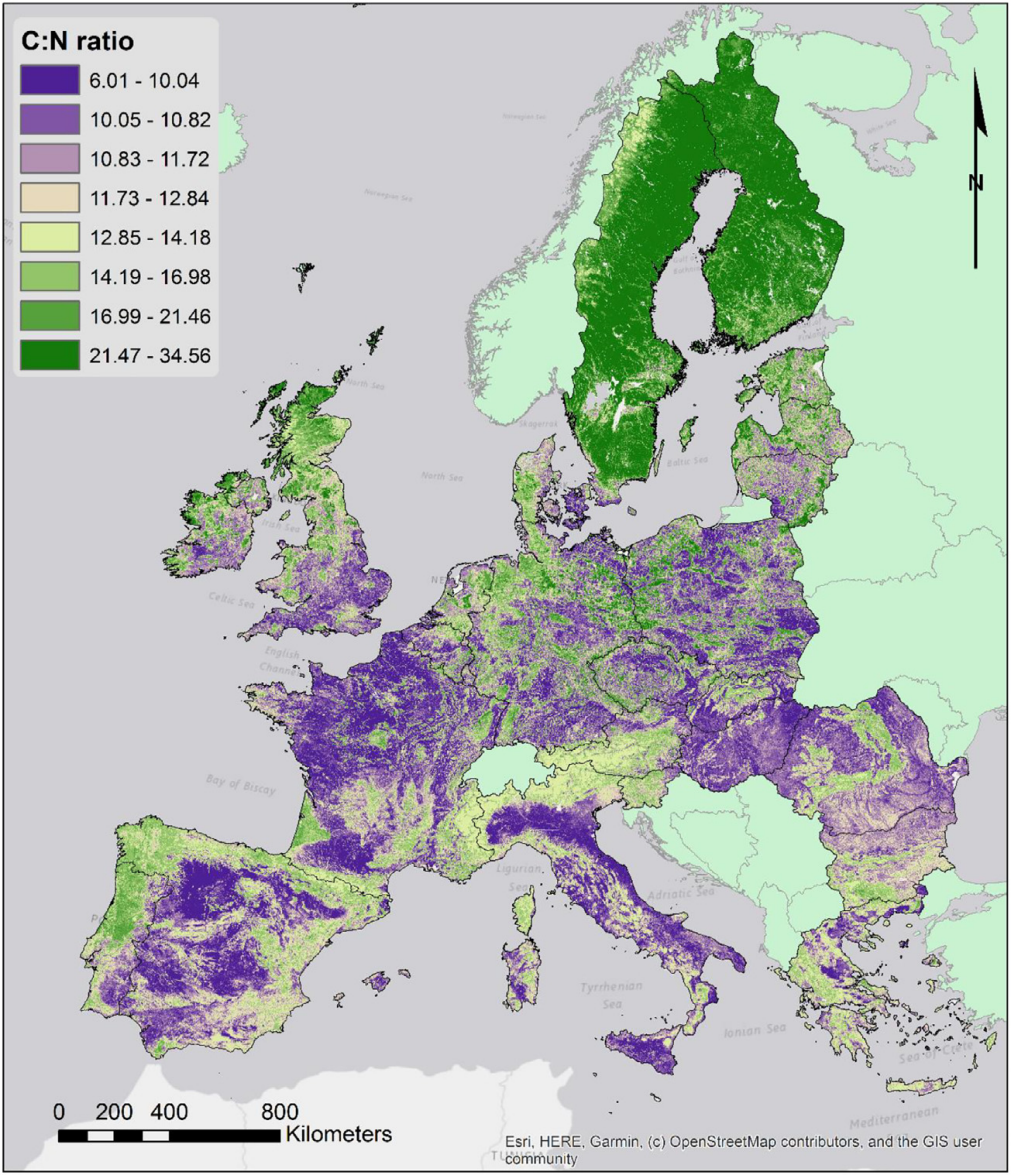
Map of the topsoil C:N ratio.

The map of the C:N ratio shown in Fig. 7 evidences the higher values in northern areas as well in areas of more intense rainfall. Vegetation distribution clearly influences the spatial distribution of the C:N ratio with higher values under coniferous trees and peatlands.

Surprisingly, the C:N ratio is better predicted (in terms of model error) by the GPR model than the other properties. This is likely due to the ratio being determined mostly by the vegetation type and not by other variables such as soil age and the geological makeup.

#### 4.2.4 Topsoil phosphorus and potassium

**Fig. 8:**
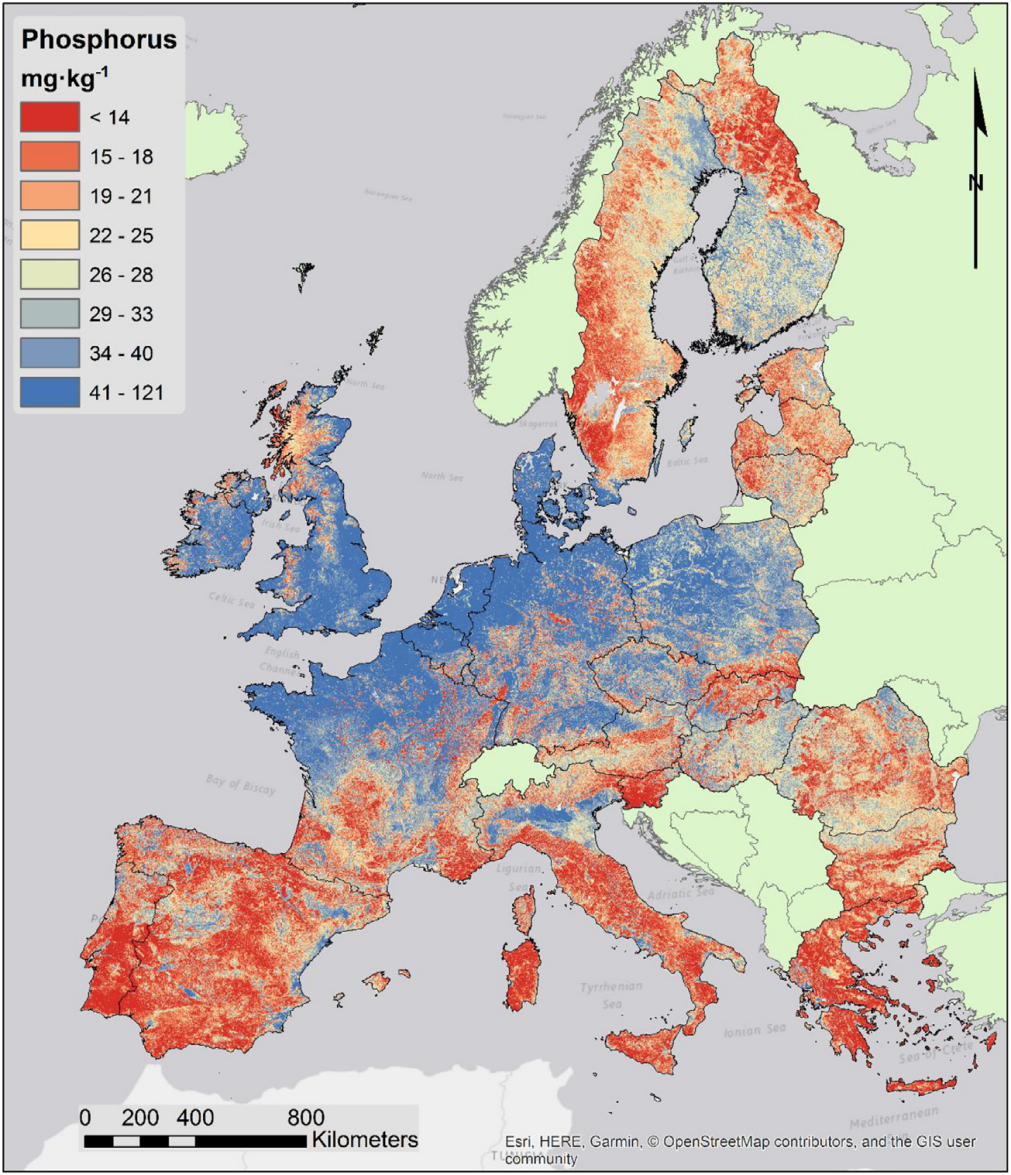
Map of topsoil phosphorous content.

The map of soil P (Fig. 8) shows a clear trend where land use appears to have a strong influence. In particular, most of the agricultural areas have higher levels of P. This is quite evident in areas like the River Po plain (Italy) where levels of P diverge from the national average. In general, areas with natural land cover and those with a prevalence of permanent crops correspond to lower levels of P.

The geological background seems to have a quite small influence, whereas climate is much more relevant. This is likely due to higher fertilization rates in relatively wetter climates. The P map produced in this study confirms models of P fertilization load (Potter et al., 2010).

**Fig. 9:**
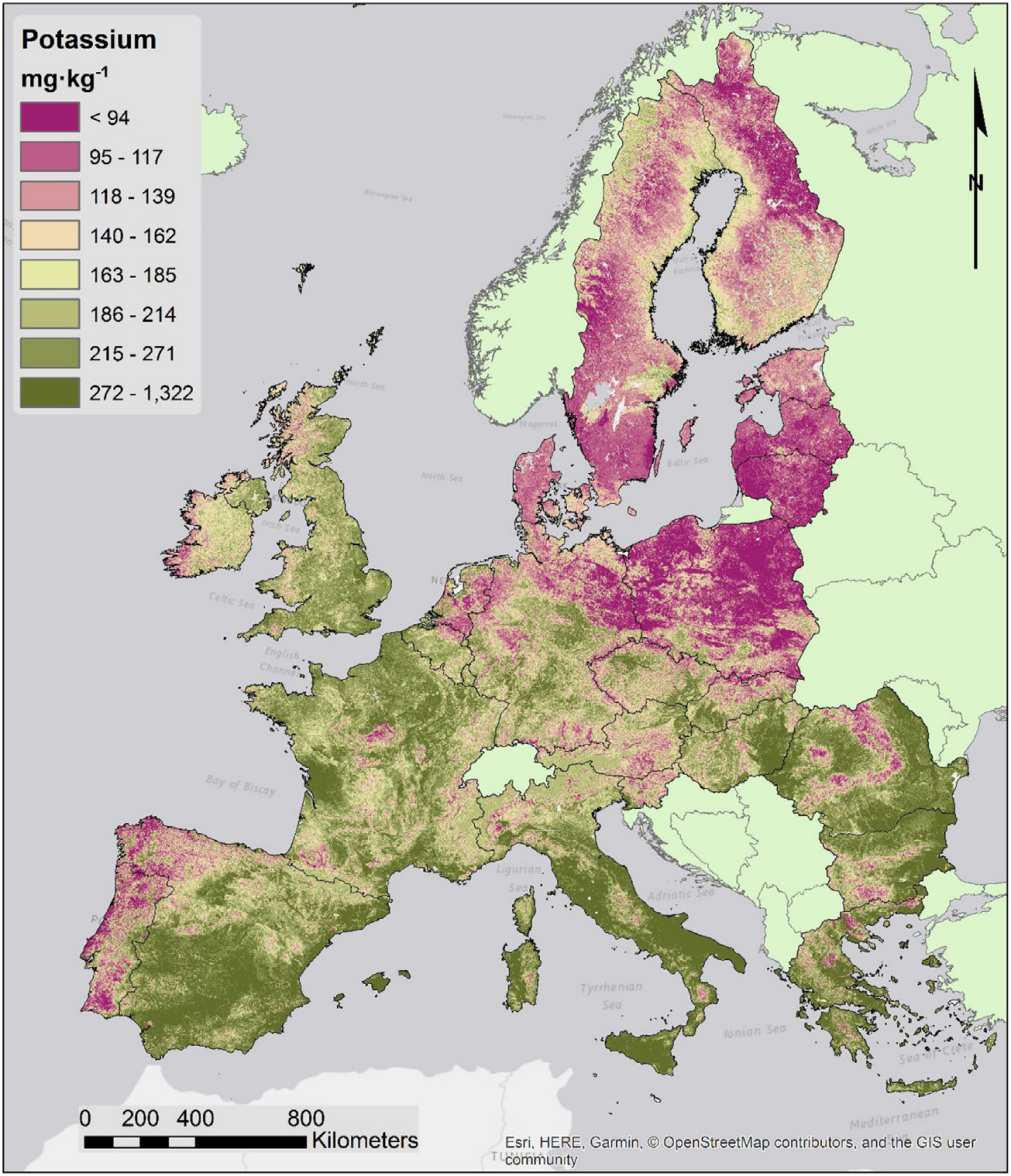
Map of topsoil potassium content.

Soil potassium distribution (Fig. 9) is mostly driven by parent material chemistry and climate. In particular, lower than average K concentrations are typical of the sandy soils of northeastern Europe, and of the relatively young soils of Scandinavia. Moreover, Portugal and northwestern Spain also exhibit lower levels of potassium likely due to leaching. In general, soils with higher clay content are better able to retain K, so the two variables show a similar spatial distribution (Ballabio et al., 2016).

### 4.3 Prediction uncertainty

The GPR predictive variance is a measure of uncertainty in the model prediction. Knowing the prediction uncertainty can be important when making predictions for areas characterised by different covariates values, compared to input data. This is analogous to kriging variance. However, while kriging variance is based on geographic distance, GPR variance is a function of the kernel distance in the covariates feature space. So while the patterns of kriging variance can be guessed by the final user from the spatial distribution of the observations, GPR variance cannot be easily assessed as it only partially depends on the samples spatial coverage.

**Fig. 10:**
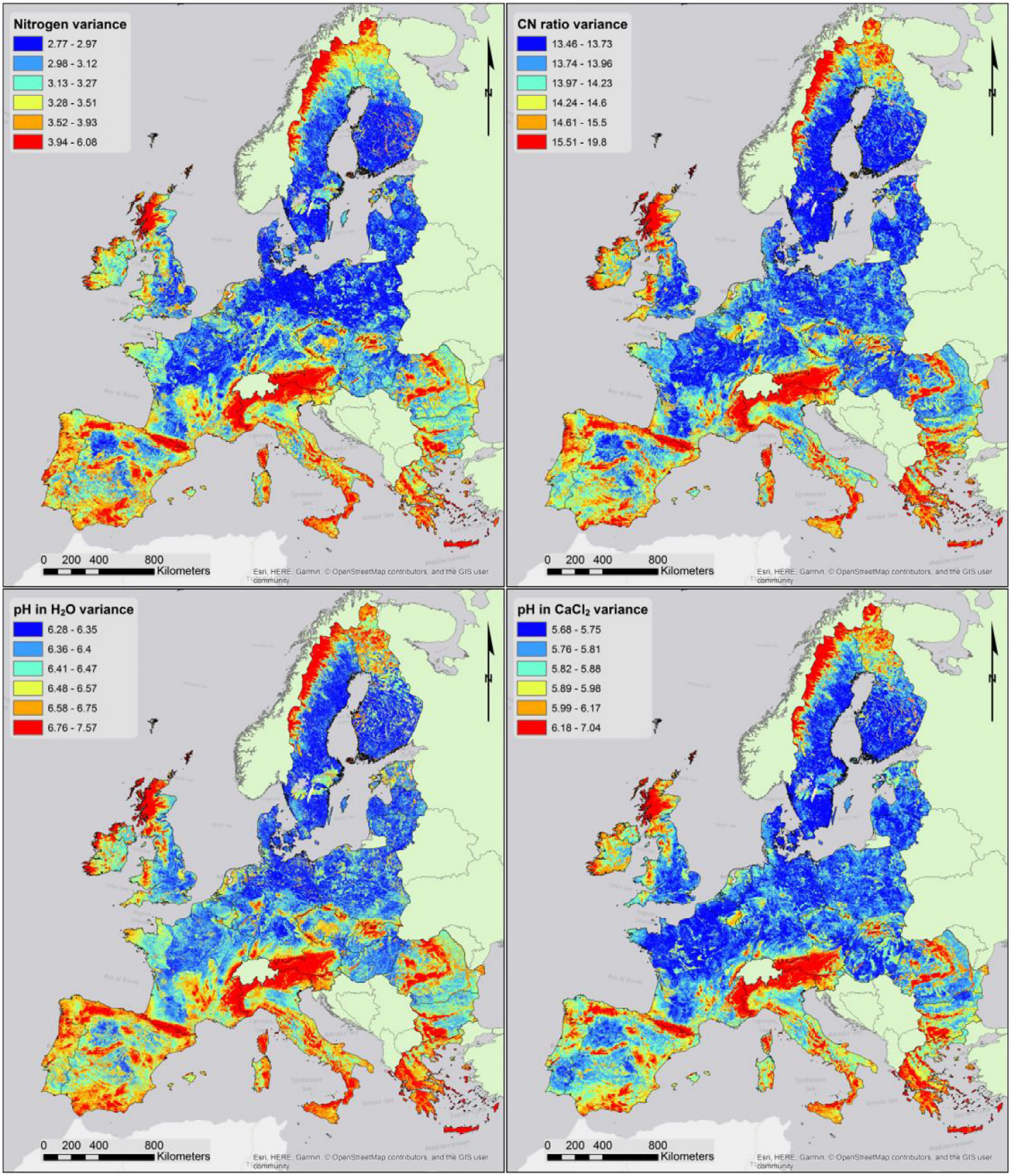
GPR prediction variance for nitrogen, C:N ratio, pH in water and CaCl_2_ 0.01 M solution.

Fig. 10 shows the prediction variances for pH, in water and CaCl_2_ solution, nitrogen and C:N ratio. While only these variables are shown, maps of variance where produced for every chemical property. As expected, some of the areas with the highest variance values are associated with unsampled areas (i.e. mountain areas above 1000 m a.s.l.). Forest areas also tend to exhibit a relatively higher variance as do areas where the presence of organic soils is more likely (i.e. Scotland and Ireland). A similar behaviour is visible in the pH maps; it is worth noting that pH measured in water has a generally higher variance than pH in CaCl_2_; this reflects the less stable measure of pH in water.

### 4.4 Data availability

The soil physical properties maps (Ballabio et al., 2016) were made available through ESDAC in September 2015. According to statistics derived for a review of LUCAS data (Orgiazzi et al., 2018), the physical properties are among the most requested datasets with almost 850 logged requests during a three year period (2015–2018). The proposed chemical properties datasets will be available in ESDAC with the publication of this study. The availability of data is an important obstacle for modellers as high spatial resolution datasets are not generally freely accessible.

## 5 Conclusions

This study provides a new set of maps of baseline topsoil chemical properties at 250 m resolution for twenty-six countries of the EU, covering an area of more than 4.5 million km^2^. The modelling is based on Gaussian Process Regression technique that allows the estimation of prediction uncertainty. The best performing prediction was obtained for the C:N ratio (R^2^ = 0.91), followed by phosphorus and potassium (R^2^ = 0.75). The performance prediction of the rest of chemical properties in terms of R^2^ is higher than 0.60 with the exception of CEC (R^2^ = 0.35).

Topsoil pH is influenced by soil parent material, erosional effects, climate and vegetation. The calcium carbonate content is highly correlated with pH, having similar influencing factors. Soil nitrogen distribution is dependent on soil organic carbon, vegetation and climate and soil texture. The land use appears to be the main driver for phosphorus content in soils, as agricultural areas have higher concentrations due to fertilizer application. CEC is influenced by the clay distribution in soils, topography and parent material.

The main limitations of the study are the number of points and the quality of some input covariates.

With 22,000 sampled locations the LUCAS soil database is unique in Europe for the number of available observations, its spatial coverage and its temporal resolution. Moreover, LUCAS soil will be improved by the additional samples taken in the 2015 and 2018 campaigns. However, at present the limitation of unsampled areas in mountains higher than 1000 m makes the prediction highly uncertain in those areas. This limitation was removed in the 2015 survey.

The resolution of the available geological covariates is very coarse and their influence in the prediction of chemical properties was limited. On the contrary, the vegetation covariates were the most significant, which means that better vegetation products such as the ones derived from the EU-ESA Copernicus program can further improve the derived chemical property maps in the future.

While LUCAS point data are available upon request from the European Soil Data Centre (ESDAC), the interpolated maps of chemical properties offer a better overview of the distribution of soil chemical properties in the EU to the scientific community and to policy makers.

The chemical properties datasets, together with the physical properties, contribute to one of the main objectives of the GlobalSoilMap project (Arrouays et al., 2017), which is to combine worldwide predictions of soil properties towards a first product of GlobalSoilMap.
